# Actin-Dependent Alterations of Dendritic Spine Morphology in Shankopathies

**DOI:** 10.1155/2016/8051861

**Published:** 2016-10-04

**Authors:** Tasnuva Sarowar, Andreas M. Grabrucker

**Affiliations:** ^1^WG Molecular Analysis of Synaptopathies, Neurology Department, Neurocenter of Ulm University, 89081 Ulm, Germany; ^2^Institute for Anatomy and Cell Biology, Ulm University, 89081 Ulm, Germany

## Abstract

Shank proteins (Shank1, Shank2, and Shank3) act as scaffolding molecules in the postsynaptic density of many excitatory neurons. Mutations in SHANK genes, in particular SHANK2 and SHANK3, lead to autism spectrum disorders (ASD) in both human and mouse models. Shank3 proteins are made of several domains—the Shank/ProSAP N-terminal (SPN) domain, ankyrin repeats, SH3 domain, PDZ domain, a proline-rich region, and the sterile alpha motif (SAM) domain. Via various binding partners of these domains, Shank3 is able to bind and interact with a wide range of proteins including modulators of small GTPases such as RICH2, a RhoGAP protein, and *β*PIX, a RhoGEF protein for Rac1 and Cdc42, actin binding proteins and actin modulators. Dysregulation of all isoforms of Shank proteins, but especially Shank3, leads to alterations in spine morphogenesis, shape, and activity of the synapse via altering actin dynamics. Therefore, here, we highlight the role of Shank proteins as modulators of small GTPases and, ultimately, actin dynamics, as found in multiple* in vitro* and* in vivo* models. The failure to mediate this regulatory role might present a shared mechanism in the pathophysiology of autism-associated mutations, which leads to dysregulation of spine morphogenesis and synaptic signaling.

## 1. Introduction

Like other eukaryotic cells, neurons have an extensive network of cytoskeletons. Among them, actin is a key player in the development of neurons and maintenance of neuronal physiology. In developing neurons, actin provides the structural network for morphogenesis. In adult neurons, actin participates in the formation and dynamics of pre- and postsynaptic structural integrity [1]. Therefore, it is not a surprise that, in many neurodevelopmental and neurodegenerative disorders, actin structure and dynamics are altered. For example, in case of autism spectrum disorders (ASD), a dysregulation of scaffolding proteins as well as receptors, signaling molecules, small GTPases, and actin dynamics in the postsynaptic density (PSD) is observed [2]. Shank (SH3 domain and ankyrin repeat containing protein) proteins (alternatively known as ProSAP, proline-rich synapse associated protein), a family comprised of 3 members, are major scaffolding proteins found in PSDs of many excitatory (mainly vGluT1 positive) synapses [3] and have been associated with ASD [4]. Mutation or deletion of Shank proteins leads to alteration in NMDA and AMPA receptor trafficking, actin remodeling, and/or alteration in synaptic signaling, in particular mGluR5 signaling, in several* in vitro* and mouse models [5]. In this review, we will focus on the effect of Shank mutation and/or deletion on synaptic spine morphology via altering actin remodeling. Interestingly, several interaction partners of Shank proteins are able to alter the spine and actin dynamics. The dysregulation of this Shank interacting complex may be a factor shared between different disease-associated mutations found in Shank3 and explain some of the synaptic phenotypes observed across different mouse models for Shankopathies. Overall, this review highlights the role of Shank in synaptic actin signaling using different signaling molecules in health and disease.

## 2. Actin Dynamics at Synaptic Terminals

### 2.1. Basic Actin Dynamics

**Figure 1:**
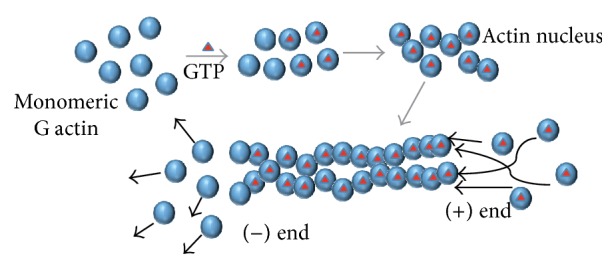
Formation of filamentous actin. Monomeric G actin can exchange GDP with GTP, depending on the energy status of the cell. Such GTP bound actin proteins are more stable and can form oligomers using weak noncovalent interactions. Such oligomers serve as nucleus for further oligomerization. Actin binding proteins (ABPs) can accelerate this process. Once oligomerized, the structure has polarity for adding new activated monomers. The (+) end can elongate the filamentous structure via adding new monomers, whereas on the (−) end the GTP bound actin is converted to GDP bound stage and dissociates from the filament. Such actin tread milling is a key component in regulation of cellular structure, morphogenesis, and activity.

Actin is a 42 kDa protein which can remain in two states—monomeric G (globular) actin and polymeric F (filamentous) actin. At any given time, some monomeric G actins exchange their ADP with ATP, thereby creating stable G actin oligomers via weak noncovalent interactions. Such oligomers act as an actin nucleus. The actin nucleus is a stable form of multimeric G actins. Formation of an actin nucleus is the rate-limiting step in the polymerization of actin since actin dimer intermediates are very unstable and addition of actin monomers to the nucleus may be prevented by actin monomer sequestering proteins [6]. Some other proteins can also work as nucleation factor such as Arp2/3. The actin nucleation center is a polar oligomer, where more actin monomers can bind with the (+) or “barbed end.” The barbed end acts as site for the biochemical reactions necessary for addition of monomers. In a cellular context, actin monomers are excess. Because of the abundance, there is no direct competition for monomers between different actin filaments at any given time. Therefore, the ongoing dynamics at the barbed end are crucial for regulation of actin polymerization, and a wide range of proteins are associated with this process. For example, some of these proteins act as capping proteins, which bind with the barbed end and prevent further addition of an actin monomer to that end [7]. On the other hand, at the (−) or “pointed end,” the ATP is hydrolyzed and monomeric G actin leaves the nucleus in its ADP bound state. Such addition and deletion of monomers create “actin tread milling” in the cell (Figure 1). Depending on the energy state in the cell, the actin can form protrusions at any certain direction or maintain a certain structure. Using this mechanism, actin can also create polarity in the cell structure, alter cellular morphology, transport organelles, participate in vesicle trafficking, and, overall, participate in signal transduction.

Besides forming filamentous structures, actin monomers can bind with many other proteins (ABPs, or actin binding proteins) in order to form higher cross-linking structures or modify the stability of different ends. For example, alpha-actinin assists monomeric G actins to cross-link with each other. EPS8 (epidermal growth factor receptor pathway substrate 8) and Gelsolin can cap the barbed end, thereby stabilizing it. Some proteins can also destabilize actin polymers like cofilin [8–10]. Several toxins have been isolated from microorganism that can influence dynamics of actin. Phalloidin, for example, can bind with F actin and enhances polymerization, whereas cytochalasins bind with the barbed end to prevent the addition of monomers [11].

### 2.2. Actin in Presynaptic Terminals

**Figure 2:**
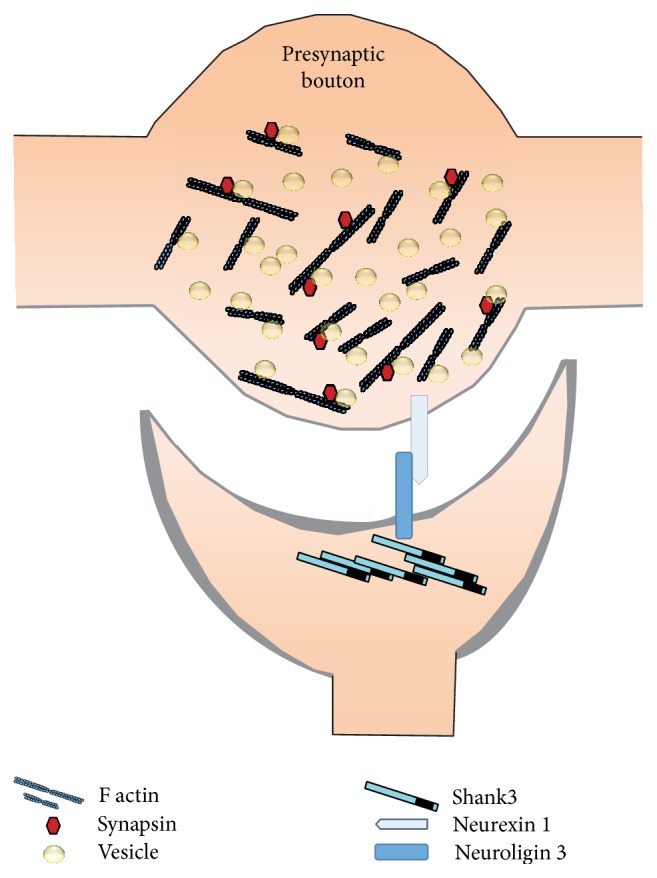
Actin in the presynaptic bouton. Actin performs a crucial role in the vesicle trafficking, starting from docking of the vesicle to neurotransmitter release. A special actin binding protein, synapsin, assists actin in this regard. Synapsin can bind with filamentous actin, and together they provide the supportive network for vesicle release. Shank3 can bind with the C-terminal tail of neuroligin 3 and thereby is able to modify the presynaptic signaling via transsynaptic neuroligin-neurexin pathways. Synaptic activity increases the amount of Shank3 in the PSD, which leads to a coordinated increase in presynaptic synapsin. Further, during neuronal development, Shank3 can also be located presynaptically, thereby directly influencing actin dynamics there.

Upon neuronal activation, neurotransmitters are released from the axon of the presynaptic neurons in the synaptic cleft. This happens via the exocytosis of synaptic vesicles at the active zone. The process of exocytosis requires calcium signaling and is achieved via several steps: docking of the synaptic vesicle to the active zone, assembly and maturation of the fusion machinery, calcium influx triggered by action potentials, and finally the fusion event [12]. Actin is abundant in the presynaptic protein pool and performs very active roles in vesicle trafficking [13–15]. It can restrict or enhance the mobility of the synaptic vesicles. At the presynaptic bouton, actin can bind with synapsin, which is phosphorylated in case neuronal activity increases (Figure 2). This actin-synapsin-vesicle interaction is very crucial for the organization of vesicles between the reserve pool and the readily releasable pool [16–19]. Through this and other mechanisms, actin can mediate synaptic efficiency. For example, actin can facilitate vesicle release from the bouton by modification of bouton size and vesicle recycling after exocytosis that requires rapid actin turnover [20].

Despite being a postsynaptic protein, Shank3 may indirectly also affect the presynaptic actin-synapsin signaling pathway mediated by transsynaptic activity involving neurexin-neuroligin protein complexes [21]. Shank3 can interact with the cytoplasmic tail of neuroligin 3 [22]. Neuroligin 3 is a postsynaptic protein that interacts with neurexin in the presynaptic neuron and, together, they have an effect on synaptic morphogenesis [23–25]. An increase in postsynaptic Shank3 levels results in increased synapsin concentrations at the presynapse [21]. Mutations in Shank3 affect the neurexin-neuroligin transsynaptic pathway. Additionally, recently it has been published that Shank mRNA and protein can be detected on the axonal terminal of the neuronal growth cone colocalizing with various presynaptic proteins there [26]. Thus, during early neuronal development, it is possible that Shank3 also acts directly on actin signaling in the presynapse.

### 2.3. Actin in Postsynaptic Dendritic Spines

Actin has pivotal role in the structure, function, and plasticity of the postsynaptic terminal. Actin remodeling is essential in various aspects of postsynaptic signaling efficiency, ranging from receptor anchoring and trafficking to dendritic spine formation. First of all, actin can bind with scaffolding proteins in the PSD and thus can anchor receptors [27]. In fact, actin can anchor AMPA and NMDA receptors on the surface of the postsynaptic dendritic spine as well as reducing the number of clusters of gephyrin in inhibitory synapses [28, 29]. Therefore, actin has the potential to influence the balance between inhibitory and excitatory signals, which is a dominant feature of synaptic processing. It has been shown that actin is found in different pools at the postsynapse, and actin dynamics and turnover can be regulated via synaptic signaling [30]. Ultimately, by modulating spine morphology, actin stabilization and depolymerization have, among others, different effects on the AMPA and NMDA receptor population [31].

## 3. Shank3/Actin in Dendritic Spinogenesis

Dendritic spines are tiny protoplasmic protrusions on the surface of dendrites and are the key receiver of excitatory stimulations in neurons. Dendritic spines can vary a lot in terms of their shape and synaptic transmission efficacy. Usually, mature spines are “mushroom” shaped with a thin neck and much larger head, while immature spines are thinner protrusions with no clear head and harboring small PSDs. Immature spines are called “filopodia-like” and “thin” spines. Such morphological diversity may arise from development and neuronal activity but is also influenced by pathological conditions [32]. Under normal circumstances, bigger spines have larger PSD areas, and the greater the area, the more the receptors located at the PSD. The main cytoskeletal component of the dendritic spine is actin, especially in the PSD where complex organization of receptors, scaffolding molecules, and actin occurs [1]. Based on the turnover rate, cellular actin can be subdivided into two pools—dynamic actin and stable actin. Upon stimulation, there is a possibility of rapid remodeling of the dynamic actin pool [33]. It has been shown that long-term potentiation (LTP) favors the shift of the G/F actin equilibrium towards F actin and thus can increase the size of the spine head. This happens in three stages: first there is the immediate reorganization of the actin cytoskeleton within the spine; then, newly formed structures are stabilized; and, finally, PSD scaffolding proteins are recruited to the modulated PSD to stabilize receptors [34]. The last stage is dependent on protein synthesis to some extent. In case of long-term depression (LTD), the equilibrium shifts towards monomeric G actin and the size of the spine head is reduced [35, 36].

**Figure 3:**
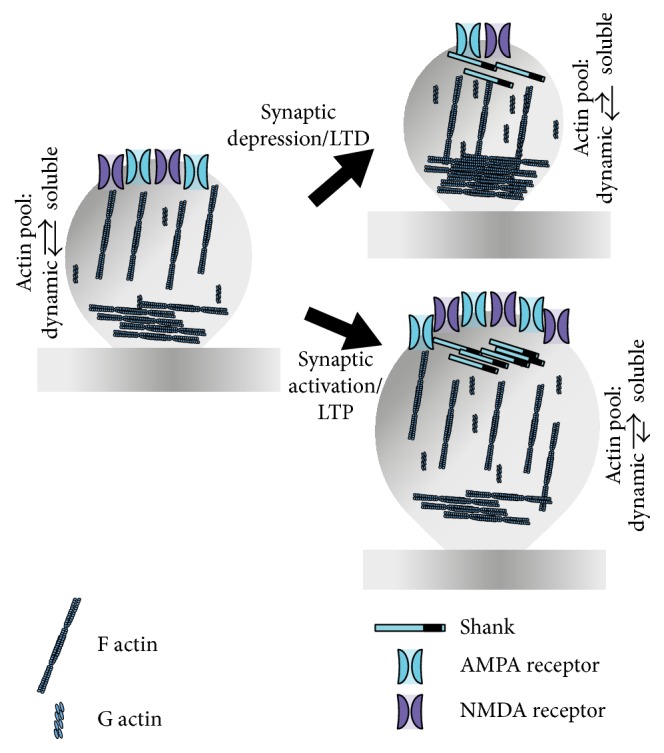
Activity dependent spine morphology alteration. Upon synaptic activation or in case of LTP, rapid actin remodeling favors the formation of filamentous or F actin. This can have twofold effects: one is the spine enlargement and the other one is that actin is able to anchor additional receptors at the PSD. On the other hand, synaptic depression or LTD is often coupled with spine shrinkage. This is realized by the depolymerization of F actin. In addition to such depolymerization, the number of receptors may be decreased, thus making the dendritic spine less responsive to synaptic stimulation. The changes in actin remodeling and receptor trafficking can occur together or independent of each other. Spine shrinkage and enlargement also depend on scaffolding proteins of the Shank family that increase in number at the PSD upon LTP and decrease upon LTD.

There is heterogeneity in the distribution of NMDA and AMPA receptors on the dendritic spine. NMDA receptors are abundant on spines irrespective of their morphology, whereas AMPA receptors are mostly present on larger excitatory spines. Therefore, it can be hypothesized that most small spines are “silent,” due to the Mg^2+^ blocking of NMDA receptors, in terms of the response towards excitatory signals; larger, mature spines can receive and enhance such inputs in much more efficient way [37]. This indicates that both the morphology of the spine and number of receptors at the PSD can be altered in response to synaptic signaling via actin remodeling (Figure 3). The modification of the actin cytoskeleton upon synaptic activity is realized by a signaling complex that transforms synaptic activity into activation of postsynaptic pathways. Here, Shank3 and small GTPases are in the focus.

Shank proteins were originally identified in the rat hippocampus in the 1990s [38, 39]. They are relatively large multiple domains proteins with more than 2000 residues and about 200 kDa in molecular mass. Because of the presence of many domains, Shank proteins are able to interact with many other synaptic proteins. Additionally, due to the SAM domain, Shank2 and Shank3 are sensitive to local zinc signaling [40–42]. It has been shown that the position of Shank is in the deeper part of PSD, probably below the PSD-95 scaffold [43].

Three isoforms of the Shank proteins have been identified: Shank1, Shank2, and Shank3 [44]. Different isoforms share 63–87% homology in their sequences. In adult rat, Shank1 is only expressed in the brain, Shank2 is expressed in brain, kidney, and liver, and Shank3 is predominantly expressed in brain, spleen, and heart [45]. In the brain, Shank1 is expressed in hippocampus, cortex, amygdala, substantia nigra, and thalamus, but not in cerebellum, caudate nucleus, and corpus callosum [46]. Shank2 and Shank3 share a common expression in different brain regions, including hippocampus and cortex. But, in cerebellum, Shank2 is expressed in Purkinje cells, whereas Shank3 is expressed in granular cell layer [47].

The various domains of Shank proteins interact with different PSD proteins and thus can link with NMDA and AMPA receptors, metabotropic glutamate receptors (mGluRs), and actin [48, 49]. It has been shown that all the Shank isoforms, Shank1, Shank2, and Shank3, upon overexpression, induce the early maturation of spines in young developing neurons [50]. In adult matured neurons, Shank1 overexpression increases the size of the spines [50].

### 3.1. Regulation of Small GTPases and Actin

The large superfamily of small GTPases consists of five subfamilies: Ras, Rho, Rab, Sarl/ARF, and Ran. These small GTPases are involved in a very diverse array of cellular actions ranging from cytoskeletal rearrangements, cellular motility, and adhesion to cellular division. Most of the studies involving small GTPases are done in nonneuronal cells. However, in the arena of dendritic spine morphogenesis, members of the Rho and Ras superfamily exhibit very distinct functions compared to other family members [51]. There are at least 14 members in the Rho family. Among them, Rac1 (Ras-related C3 botulinum toxin substrate 1) and RhoA (Ras homolog gene family, member A) are the most studied family members. In dendritic spine morphogenesis, Rac1 and RhoA have opposite effects [52, 53].

**Figure 4:**
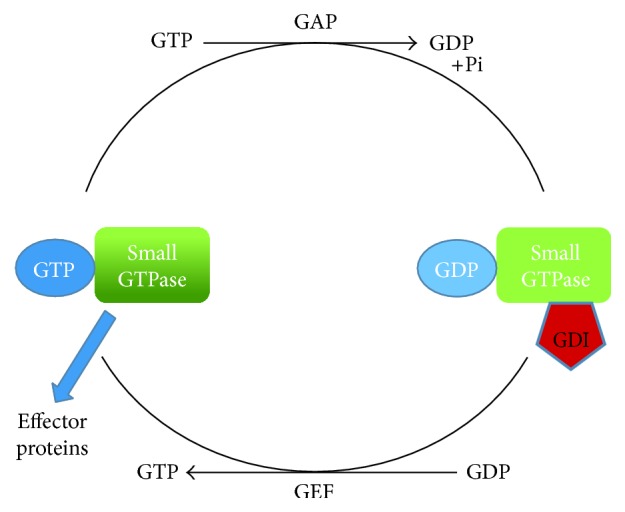
Small GTPases act like a molecular switch. Small GTPases are active when they are GTP bound and inactive when they are GDP bound. The switch between active and inactive state is mediated by modulators, mainly GAPs (GTPase activating proteins) and GEFs (guanosine exchange factors). GAP hydrolyzes the bound GTP to GDP and GEF exchanges the GDP with GTP. Besides GAPs and GEFs, there is another kind of modulators—GDI (GDP dissociation inhibitors).

Small GTPases can act as molecular switch. They are turned “on” when bound with GTP and “off” when bound with GDP. GTPase modulators such as GEFs (guanosine exchange factors) and GAPs (GTPase activating proteins) mediate this switch (Figure 4). GEF activates the small GTPase whereas GAP enables the small GTPase to go back in the “off” state. Besides GEFs and GAPs, there is another kind of modulators, GDI (guanosine nucleotide dissociation inhibitors). When small GTPases are GDP bound and inactive, then GDI proteins sequester them in the cytoplasm [54].

Overexpression of Rac1 leads to maturation of spines, whereas overexpression of RhoA leads to spine loss [55]. The downstream effectors of Rac1 are PAK (p21 activated kinase), LIMK1 (LIM kinase 1), and actin binding protein cofilin [56, 57]. Interacting with these proteins, Rac1 stabilizes F actin. The main downstream effector of RhoA is ROCK (Rho associated protein kinase). Interestingly, ROCK can regulate the activity of LIMK1 via controlling its phosphorylation [58]. Cdc42 (cell division control protein 42) is a Rac related GTPase and effects of Cdc42 in terms of dendritic spine morphology are similar to Rac1. Besides the effect on postsynaptic spine morphology, the transformation of immature axonal boutons to mature boutons requires actin polymerization mediated via BDNF and Cdc42 activation [59].

Since the activity of small GTPases depends on their modulators, inhibition or deletion of the modulators in turn can affect small GTPase signaling. RhoA is mostly associated with spine shrinkage and actin destabilization. GEFs that activate RhoA such as RhoA GEF H1 therefore have similar effect and regulate spine density and length negatively [60]. Several GEFs for Rac1 have been studied extensively. However, in the cortex of adult mice, Kalirin-7 is the only expressed Rac1 GEF [61]. Overexpression and knock-down of Kalirin-7 have very clear-cut opposite effects on spine morphogenesis: overexpression increases spine density and head area, whereas RNAi mediated knock-down reduces both [61–63]. In hippocampus, besides Kalirin-7, two other Rac1 GEFs are expressed: Tiam1 and *β*PIX. Tiam1 interacts with NMDA receptors, and NMDA receptor activation can induce Tiam1 phosphorylation in a calcium dependent manner that activates actin remodeling dependent on Rac1 signaling in dendritic spines [64].

Besides GEFs, some GAP modulators are also well characterized in the central nervous system (CNS). Loss of the RhoGAP protein Oligophrenin 1 disrupts spine and synapse maturation [65]. Loss of the RhoGAP protein RICH2 leads to increased activation of Rac1 and Cdc42 and increases spine area in mouse model [66]. SynGAP1, a Ras GAP, can regulate spine morphology via its effect on Rho GTPase and cofilin [67].

Small GTPases can affect actin binding proteins directly. They can relieve the autoinhibition of WASP (Wiskott-Aldrich syndrome protein), which binds to both filamentous and monomeric actin [68]. Along with Arp2/3 (actin related protein2/3) and Cdc42, WASP can form a protein complex that modulates actin assembly [69, 70]. Therefore, Arp2/3 serve as downstream signaling proteins of small GTPases and WASP.

### 3.2. Shank and Spine Morphogenesis via Actin Regulation

Mutations in SHANK have very clear-cut effect on synaptogenesis and Shank can recruit other synaptic proteins such as Homer1, which results in alterations of spine morphology [50]. Shanks can bind NMDA receptors via PSD-95/GKAP and mGluRs via Homer1 [50]. Both PSD-95 and GKAP are involved in activity dependent spine growth [71]. Another interaction partner of Shank proteins is cortactin. Shank knock-down reduces both cortactin and actin in the spine in cultured rat hippocampal neurons [72].

Further, in an interactome study, it was found that Shank3 directly interacts with Arp2/3 [73]. This is a direct link between actin remodeling and Shank3. The first step of actin polymerization is the decapping of the barbed end of actin filaments so that the filament can be exposed for further addition of G actin. The Arp2/3 complex helps in this regard. The Arp2/3 complex consists of seven subunits including two actin related proteins—Arp2 and Arp3 [74]. The Arp2/3 complex lowers the affinity of capping proteins for barbed ends [75], which enables decapping, and further monomers can be added to the filament. Additionally, Arp2/3 helps to create complex multibranched actin filaments. The activity of Arp2/3 can be further enhanced by other NPFs (nucleation promoting factors). Two such NPFs are WASP (Wiskott-Aldrich syndrome protein) and SCAR/WAVE (suppressor of cAMP receptor/WASP family verprolin homologs). WASP can bind with Cdc42 in a GTP-dependent manner [76]. Small actin nuclei first bind with WASP; then Arp2/3 joins the WASP-actin complex, facilitating decapping of the barbed end. Subsequently, additional actin monomers can bind with that end and the filament size is increased. Once the filamentous actin is formed, then cortactin replaces WASP and stabilizes the filamentous actin structure [77]. Thus, using Arp2/3 and cortactin, Shank3 is able to stabilize F actin and enhance spine maturation.

**Figure 5:**
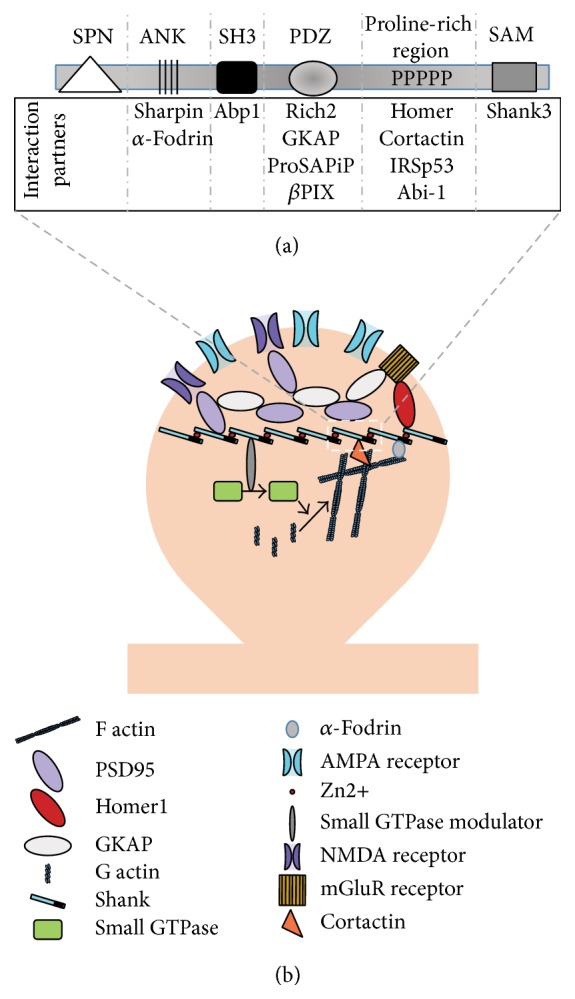
Cross talk between Shank and small GTPases. (a) The domain architecture of Shank proteins and their interaction partners. The Shank protein consists of 5 different important domains: N-terminal ankyrin repeats, Src homology domain 3 (SH3), PSD-95/Dlg/ZO-1 domain (PDZ domain), proline-rich region, and C-terminal sterile alpha motive. Different proteins can interact with different domains. For example, the C-terminus of GKAP, small GTPase modulator *β*PIX, ProSAP interacting protein (ProSAPiP), and the C-terminal STAV motif of RICH2 can interact with the PDZ domain of Shank3. Between the PDZ domain and SAM domain, there is a gap of more that 1000 residues, which is proline-rich. This proline-rich region can interact with Homer, IRSp53, Abi-1, and cortactin. The SAM domain of Shank2 and Shank3 can bind with other SAM domains upon zinc sequestration organizing platforms in an antiparallel manner. (b) Within the dendritic spine, Shanks interact with a wide range of proteins, for example, linking other scaffolding molecules like PSD-95, Homer, and GKAP and associated receptors to the actin cytoskeleton. Some of Shank's interaction partners are modulators of small GTPases; thereby they can activate actin remodeling via GTPase signaling. The interaction partners cortactin and *α*-fodrin can stabilize actin. Such actin reorganization alters the morphology and maturity of spines and strengthens synaptic transmission.

*α*-Fodrin which interacts with the ankyrin repeats of Shank via its spectrin motif can bind with F actin [78]. Further, ProSAPiP1 (ProSAP interacting protein), an interaction partner of the Shank3 PDZ domain, has an actin binding domain and thus it can influence actin remodeling [79]. In addition, the Abp1 (actin binding protein 1) is an interaction partner of Shank, which interacts with the SH3 domain [80]. Abp1 can regulate actin remodeling in the spine head and thus participates in spine morphogenesis [80]. Finally, besides Homer1 and cortactin, Abi-1 (Abelson interacting protein 1) interacts with the proline-rich region of Shank3. Abi-1 can modulate spine morphology and synapse formation [81] (Figure 5(a)).

Shank proteins also interact with SPIN90 (SH3 protein interacting with Nck, 90 kDa), which plays an important role in actin polymerization, endocytosis, growth cone formation, and dendritic spine morphogenesis [82]. SPIN90 is phosphorylated by Src kinase [83], which leads, similar to overexpression of SPIN90, to enlargement of spines, while SPIN90 knock-out in neurons results in an altered actin cytoskeleton [83]. Further, neural Abelson-related gene-binding protein 2 (nArgBP2), a protein that interacts with SAPAP3 and Shank3, was found to regulate spine morphogenesis through the activation of the Rac1/WAVE/PAK/cofilin pathway [84].

Taken together, these interactions provide a strong link between Shank proteins and actin regulating signaling pathways. Within the PSD, Shank3 is concentrated preferentially in the distal layer after synaptic activity and in presence of Zn^2+^ [43]. It has been hypothesized that subsequent recruitment of Shank binding proteins such as IRSp53, Abp1, and cortactin to the distal layer could mediate acute regulation of the actin cytoskeleton in response to synaptic activity [85]. Thus, mechanistically, Shank proteins might be in the center of a PSD actin modulatory complex, regulating synaptic actin dynamics via several central pathways.

### 3.3. Shank Signaling Affecting Small GTPases

Some interaction partners of Shank proteins act as modulators for small GTPases. One such interaction partner of Shank3 is RICH2 (RhoGAP interacting with CIP4 homolog 2). The RICH2 protein interacts with the PDZ domain of Shank3 via its C-terminal STAL/STAV motif.* In vitro* studies show that, during LTP, the Shank3-RICH2 interaction is increased and localized at the dendritic spine. Additionally, RICH2 controls AMPA receptor trafficking and spine morphology [86]. As RICH, the firstly identified homolog of RICH2, RICH2 was identified as a GAP protein for Rac1 and Cdc42 [87]. In the RICH2 knock-out mouse model, the brain size was increased, synaptic NMDA receptor levels were increased, and the spine area was enlarged. Further, a high number of fused multiple spine synapses were detected [66]. Moreover, in this mouse model, Rac1 and Cdc42 were identified as the candidate small GTPases on which RICH2 can exert its RhoGAP activity. Deletion of RICH2 leads to constitutive active signaling of Rac1 and Cdc42, which mediate actin polymerization. Interestingly, this mouse model showed a subset of autistic symptoms on behavioral level such as an increase in stereotypic movements. Additionally, RICH2 knock-out mice had a specific fear of novelty in terms of novel objects.

*β*PIX, a RhoGEF for Rac1 and Cdc42, can interact with the PDZ domain of Shank using its leucine zipper domain and PDZ binding domain [88]. Shank can bind with *β*PIX and *β*PIX associated signaling molecules like PAK (p21-associated kinase). Since PAK is a downstream signaling molecule of Rac1 and Cdc42, such interaction can affect small GTPase signaling and actin remodeling [51]. Deletion of *β*PIX in drosophila leads to neuromuscular junction defects and reduction of synaptic proteins including PAK and glutamate receptor [89].

IRSp53 (insulin receptor tyrosine substrate kinase 3) interacts with the proline-rich region of Shank3. IRSp53 is an insulin receptor substrate in the brain and acts in the downstream signaling of the small GTPase Cdc42. In this way, IRSp53 modulates the organization of spines and actin [90]. Besides Shank3, IRSp53 can also interact with PSD-95 and recruit PSD-95 to the spines [91]. Thus, IRSp53 can form a tripartite complex with Shank and PSD-95, linking again the spine morphogenesis and small GTPase signaling.

Some modulators of the small GTPases are also indirect interaction partners of Shank proteins. One such modulator is SPAR. SPAR can interact with the PDZ domain of Shank using ProSAPiP1 as linker molecule [92]. It was first identified in a yeast two-hybrid screen using the guanylate kinase (GK) domain of the PSD-95 family member PSD-93 [93, 94]. SPAR acts as a GAP protein for Rap small GTPases. SPAR has two actin interacting domains and it colocalizes with PSD-95 in dendritic spines of hippocampal neurons in culture. Overexpression of SPAR leads to multilobed shaped dendritic spines. Moreover, SPAR can reorganize F actin into large aggregates [93].

Thus, there is significant overlap between the Shank mediated action on dendritic morphology and small GTPase mediated signaling pathways targeting actin dynamics. It is thus possible that Shank signaling at synapses intersects with these pathways, which drive the formation, maturation, and activation of dendritic spines (Figure 5(b)). The receptors that are mainly responsible for synaptic plasticity-driven spine remodeling are NMDA receptors. Shank proteins act as a scaffold, holding other scaffolding molecules like Homer1, GKAP, and PSD-95, thus facilitating the recruitment of NMDA receptors. NMDA receptors in turn can regulate small GTPase modulators like Kalirin-7 using various mechanisms like phosphorylation and calcium dependent kinases.

## 4. Shank and Small GTPase Modulators in Neurological Disorders

**Figure 6:**
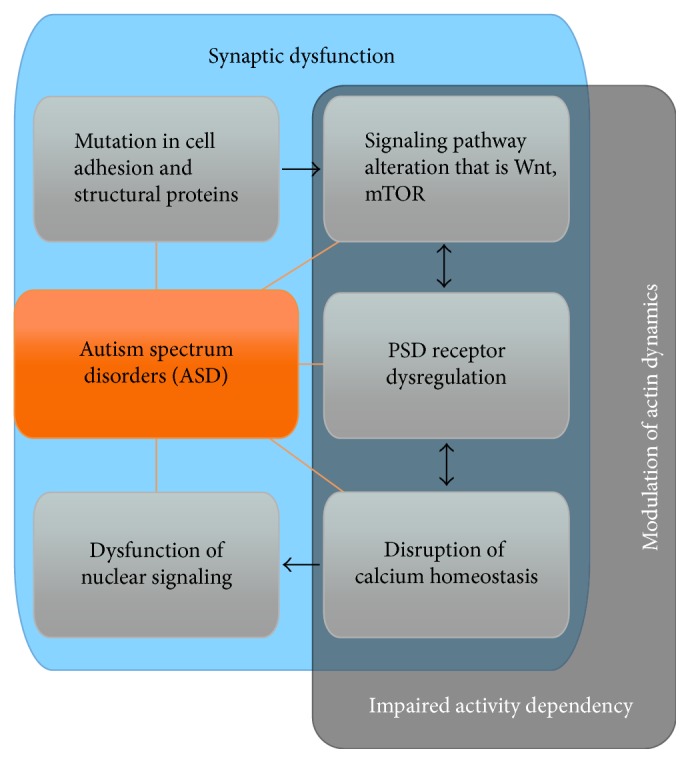
ASD associated pathways. In very few cases, ASD is caused by the dysfunction of a single gene, for example, in syndromic forms of autism such as Rett syndrome (mutation in MeCP2 gene), Angelman syndrome (UBE3A gene), and fragile X syndrome (FMRP1 gene). In nonsyndromic forms, mutations in PSD adhesion molecules like neurexin and neuroligin and structural proteins like Shank are frequently found at excitatory synapses. This may lead to mGluR5 or NMDA receptor dysregulation (i.e., GRIN2A/B) and ultimately, among others, calcium homeostasis imbalance, disruption in signaling pathways like Wnt or mTOR signaling, and dysfunction of nuclear signaling. Underlying or accompanying all these alterations are impairments in action dynamics within dendritic spines.

ASD are identified as a term collectively describing various similar neurological disorders. The core features of ASD are language and communication impairment, deficits in social interaction, and repetitive stereotypic behaviors [95]. In addition to these, there are many other psychological disturbances like anxiety, sleep disturbances, and epilepsy, as well as comorbidities such as gastrointestinal abnormalities. ASD are considered as heritable disorders often with environmental factors acting on a specific genetic background to trigger the disease, and till now more than 100 genes have been associated with ASD [96, 97]. The range of synaptic proteins that has been associated with ASD is quite diverse. They include NMDA receptors (GRIN2A, GRIN2B, and GABRB3), master PSD scaffolding proteins (SHANK2, SHANK3, and glutamate receptor interacting protein 1), PSD adhesion molecules (Contactin 4, Contactin associated protein like-2/4, neurexin 1, neuroligin 3, and neuroligin 4), and hormonal receptors (oxytocin receptor) [97]. It has been hypothesized that mutation in synapse associated proteins produces mutated/truncated proteins that are unable to perform their normal physiological functions, which ultimately leads to circuit dysfunction and finally behavior abnormalities [97]. Surprisingly, many of these ASD candidate genes intersect at common pathways at synapses (Figure 6). Such pathways, for example, control synapse formation and receptor trafficking. Actin remodeling is a major contributor to these processes.

Given the central role of SHANK in disorders such as ASD, it is likely that impairment in these processes is a major contributor to the phenotypes observed after mutation or deletion of SHANK genes. Recently several modulators of GTPase have been found to be associated with neurological disorders, due to the profound effect of small GTPases on dendritic spine morphogenesis. Most common of these is intellectual disability. Mutation in oligopherinin 1, a RhoGAP for RhoA, Rac1, and Cdc42, leads to reduction in spine length and number of mature spines accompanied by resulting phenotypes including intellectual disability, epilepsy, microcephaly, ataxia, and hyperactivity [98, 99].

Dysregulation of MEGAP, another RhoGAP for Rac1 and Cdc42, has also been associated with intellectual disability and microcephaly. Additionally, the individuals have long faces and long ears [100]. MEGAP interacts with Homer, which is a direct interaction partner of Shank [101]. Two GEF modulators, ARHGEF6 (or *α*PIX) and Alsin, have been identified as the causative genes for intellectual disability and motor neuron degeneration [102–106]. Mutation in Alsin, a GEF for Rac1, Rab5, and Ran, leads to increased neuronal death and reduced axon growth, whereas mutation in ARHGEF6, a GEF for Rac and Cdc42, decreases the amount of mature mushroom type spines [107].

Besides modulators, some effector molecules have been identified as causative genes for intellectual disability. Examples include PAK3, LIMK1, and FMRP (interacting with the downstream effector of Rac1, CYFIP). Mutation in PAK3 alters synaptic efficacy via impairing LTP and decreasing mature synapses [108]. FMRP (fragile X mental retardation protein) is most commonly associated with fragile X syndrome. It is also an indirect downstream effector of Rac1 [109, 110]. Further, the Rap1 GEF, Epac2, has been associated with ASD [111–113]. Mutations in SynGAP1, a Ras GAP, have been found in a patient suffering from ASD and intellectual disability [114–117]. Intriguingly, mutations in SHANK3 are highly associated with intellectual disability and are especially abundant in patients suffering from ASD with moderate to profound intellectual disability [118].

Taken together, different mutations in small GTPase effectors and modulators have been associated with mental abnormalities (Table 1). Small GTPase modulators and effectors are able to create phenotypic alteration in human via modifying spine morphology [107] which were also observed in SHANK knock-out mouse models.

## 5. Abnormal Actin Remodeling in Shankopathies

Identification of Shank3 mutation (22q13.3 deletion, Phelan McDermid syndrome) in a patient was the first link of Shank proteins with neuropsychiatric disorders in humans [119, 120]. SHANK3 is one of the best characterized genes associated with ASD. Shank mutations may lead to other comorbidities, besides ASD, like intellectual disability, schizophrenia, bipolar disorder, and attention deficit and hyperactivity disorder (ADHD) [4, 5]. Shank3 duplication can lead to other symptoms like ADHD and bipolar disorder in human [73]. Shank mutations have been identified in almost 1% of all ASD patients [118]. The mutations vary a lot in terms of the peptide residues including whole exon deletion, whole domain deletion, and even point mutations. Mutations in Shank have very high penetrance at the phenotypic level, and any form of Shank3 haploinsufficiency is enough to cause behavioral disturbances [5].

Golgi analyses in the postmortem ASD brain tissues show that there are alterations in dendritic spine density [121, 122]. Since the Shank proteins are modulators of dendritic spine morphogenesis, it is very possible that mutation or deletion of Shank leads to alteration in spine homeostasis.

Many mouse and rat models have been generated across the globe to mimic human Shankopathies. For example, several groups studied Shank1 knock-out mice [123–125]. This mouse model had alteration in synaptic protein composition but also impairments in dendritic spine morphology; the number of dendritic spines was reduced, and the spines were thinner and smaller.

Shank2 knock-out mice that exhibit almost all the core features of ASD like increased stereotypic behavior, impairment in social interaction, hyperactivity, and anxiety [126] similarly show a reduction in the number of dendritic spines and basal synaptic transmission with depletion of NMDA receptors [126]. Allosteric mGluR5 modulators, partial agonists of NMDARs (D-cycloserine), and the zinc ionophore clioquinol rescued the phenotype by enhanced NMDA receptor activity [127, 128].

Several mouse models have been generated to investigate the role of Shank3 and its splice variants in ASD. Recently, complete knock-out of Shank3 in mice was published [129]. In Shank3*α* and Shank3*β* knock-out mice, the corticostriatal circuitry is defective and fewer and thinner PSDs were detected; in addition, the amount of receptors and scaffolding proteins was reduced. In the complete Shank3 knock-out mouse model, mGluR5-Homer scaffolds and mGluR5 signaling are impaired. Additionally, the autistic mouse model showed impaired function of striatal synapses and abnormalities in brain morphology [129].

Further, Shank3 knock-out mice were reported to suffer from a loss of cortical actin filaments. A reduced Rac1/PAK activity along with increased activity of cofilin, the major actin depolymerizing factor, has been identified as the underlying biological process for this phenotype. The aberrant regulation of synaptic actin filaments that might be connected to the loss of synaptic NMDAR observed in these animals was proposed to contribute to their autism-like behavior. Intriguingly, the social deficits and NMDAR hypofunction were rescued by inhibition of cofilin or activation of Rac1 signaling [130].

**Figure 7:**
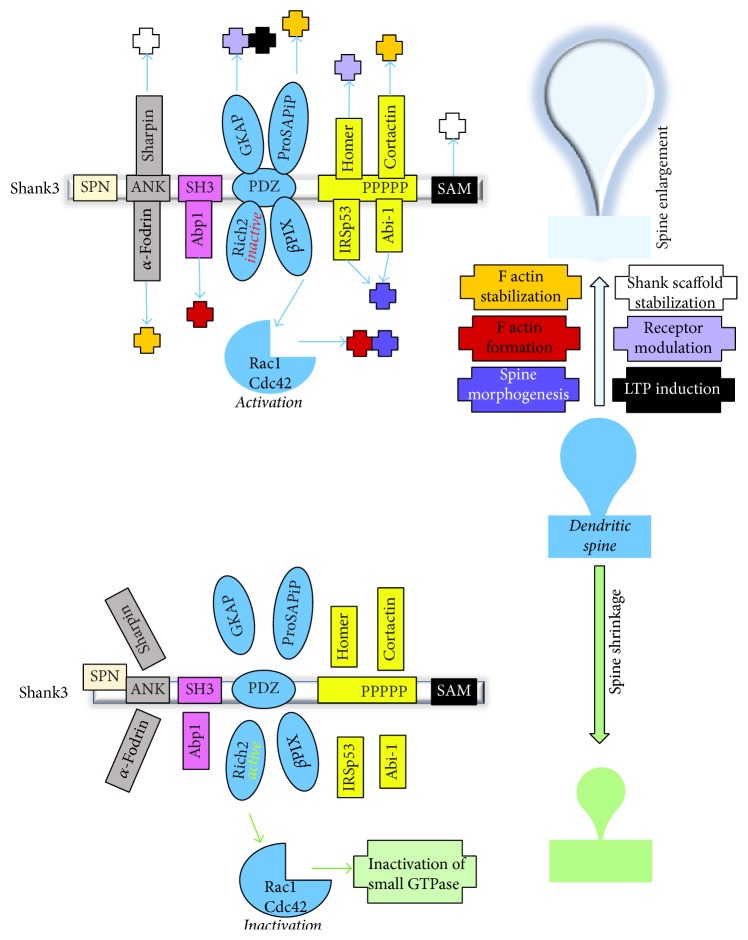
Shank3 and its interaction partners can affect actin cytoskeleton and spine morphology via various pathways. Shank3 and its many interaction partners can affect spine maturation and morphogenesis. Ankyrin repeat domain interaction partners, sharpin and *α*-fodrin, enable spine enhancement via Shank scaffold stabilization and F actin stabilization. SH3 domain interaction partner, Abp1, enhances F actin formation. Other scaffolding molecules, GKAP and Homer, can interact with Shank3 and take part in AMPA, NMDA, and mGluR modulation. Small GTPase modulator like *β*PIX activates Rac1 and/or Cdc42 and enables F actin formation and spine morphogenesis. Cortactin, intertion partner of proline-rich domain, binds and stabilizes F actin. Thus, most of the Shank3 interaction partners have a positive role in spine morphogenesis and mutations or deletions result in spine shrinkage and loss of synapses. An exception is Rich2. Rich2 deactivates Rac1 and/or Cdc42 and thus can take part in spine size reduction and spine loss. Shank3 bound Rich2 is inactive. Therefore, a switch between Shank3/Rich2 to Shank3 and unbound Rich2 at the PSD may be an important integrator of the translation of synaptic activity in controlled and restricted spine growth. It is mentionworthy that, at any certain time point, only one interaction partner of the same domain can bind with Shank3.

Thus, Shank proteins and their various interaction partners are able to modulate spine morphogenesis via actin remodeling in response to synaptic activity (Figure 7). Upon synaptic activation, zinc is released from the presynaptic terminal at zincergic synapses. The free zinc can bind to receptors such as NMDAR on the postsynapse or enter the postsynaptic terminal. Additionally, at zincergic but also other neurons, zinc levels may increase within the postsynapse by release from intracellular stores like metallothionein 3 upon neuronal activation, which triggers the recruitment of Shank2 and Shank3 proteins to the PSD via formation of Shank platforms providing the framework for binding of other proteins. That way, further direct and indirect interacting partners can bind to the scaffold and modulate the PSD and spine using different mechanisms [131].

To start from the N terminal, the less well known SPN domain (Shank/ProSAP N-terminal domain) has a role in the function of the ankyrin repeat domain (ANK). Recently it has been shown that the SPN domain is able to interact with the ANK domain of the same protein, thus creating an intramolecular interaction [132] and thereby reducing the accessibility of the ANK domain. Through this, interaction partners of ANK domain like *α*-fodrin and sharpin are unable to bind with Shank3. Some point mutations have been identified in the SPN domain of Shank3. Due to these mutations, the SPN domain loses its ability to bind with the ANK domain, thereby enhancing sharpin and *α*-fodrin recruitment [132].

The cytoskeletal protein *α*-fodrin interacts with the ANK domains of both Shank1 and Shank3 using its conserved spectrin motif [78]. Fodrin can bind with actin and stabilizes actin filament. Additionally, fodrin serves as a calmodulin binding protein [133]. Therefore, indirectly fodrin can link Shank proteins with the calcium sensing mechanism in the neuron. Besides *α*-fodrin, the C terminal of sharpin can also bind with the ANK domain of Shank proteins [134]. Different point mutations in the ANK domain have been identified that lead to a decrease in actin filaments [135] as well as spine density and spine maturation. In case the ANK domain is deleted or mutated, *α*-fodrin and sharpin are unable to bind Shank. This, on one hand, affects actin stabilization via *α*-fodrin and, on other hand, hampers the sharpin-Shank scaffolding. For Shank3, many mouse models have been generated to investigate its role in PSD. This list today includes at least 5 different partial knock-out mouse models, one complete knock-out model, and an overexpression mouse model [73, 126, 129, 136–139]. Two mouse models harbor deletion of exons 4–9 in the ANK domain [136, 138]. Since the ANK domain binds with sharpin and *α*-fodrin, impairment of these interaction might be an underlying cause for the alterations observed in the PSD protein composition [138]. The spine length was increased without any increase in spine head and the total number of spines was reduced.

The PDZ domain of Shank is very important in terms of its scaffolding function. Several small GTPase modulators (i.e., RICH2 and *β*PIX) and other scaffolding proteins (i.e., GKAP) bind at this domain. RICH2 acts as a RhoGAP for Rac1 and Cdc42. If RICH2 is activated, it inactivates Rac1 and Cdc42. Rac1 and Cdc42 enhance dendritic morphogenesis. Thus, activation of RICH2 is associated with spine size reduction. On the other hand, if RICH2 is deleted, both Rac1 and Cdc42 are hyperactivated leading to changes in spine shape and morphology [66]. The function of Shank3 bound Rich2 and Shank3 unbound Rich2 is opposite in* in vitro* models [86]. Upon cLTP, mutated Rich2 that is unable to bind with Shank3 inhibits spine head enlargement. This points to a role of Shank3 as inactivator of Rich2 to promote spine enlargement.

Another small GTPase regulator that binds with the PDZ domain of Shanks is *β*PIX [88]. *β*PIX works as a RhoGEF for Rac1 and Cdc42. If *β*PIX is activated, it converts the GDP bound inactive Rac1/Cdc42 to GTP bound active Rac1/Cdc42 activating the downstream signaling molecules. Unlike Rich2, if *β*PIX interacts with Shank3, it is activated, which promotes spine enlargement. Thus, binding of both *β*PIX and Rich2 may increase the activation state of Rac1/Cdc42 in the dendritic spine due to the different effects of Shank3 on the activity of these proteins [66, 88].

Peça et al. characterized a mouse model which had deletion in exons 13–16, spanning the PDZ domain of Shank3 [139]. A number of PSD proteins and receptors were reduced in this mouse model. In the striatum, several morphological abnormalities of dendritic spines were observed like a decrease in spine density, length, and thickness. All this points to the fact that the PDZ domain is important for the scaffolding function of Shank3 and for the maintenance of spine structure and function.

The proline-rich domain of Shank3 proteins is also very important for synaptic signaling as it binds cortactin, Abi-1, and IRSp53. Cortactin binds and stabilizes F actin. Abi-1 is in a complex with proteins such as WASP/WAVE, EPS8, and cortactin, thereby regulating the actin cytoskeleton via the Arp2/3 complex [81]. Knock-down of Abi-1 reduces synapse density, while it increases dendritic branching and the number of immature spines. Like Rich2 and *β*PIX, IRSp53 links small GTP signaling pathways and Shank signaling, as it acts as a downstream effector of Cdc42. Therefore, this domain, along with its interaction partners, promotes spine enlargement and maturation.

Recently a complete knock-out mouse model of Shank3 has been characterized which has deletion of exons 4–22 in Shank3 [129]. There were reductions in spine density and spine length (in striatum). A Shank3 overexpression mouse model overexpressing the major isoforms of Shank3 confirms that Shank3 can directly interact with Arp2/3 and many other actin modulators and small GTPase signaling proteins [73]. However, the actual interplay among these proteins is yet to be determined.

From these data, a model can be hypothesized, according to which synaptic activity should facilitate an open conformation of Shank3 regarding its SPN and ANK domains to enable binding of proteins promoting F actin formation and subsequent stabilization. Further, proteins such as *β*PIX should induce the activation of modulators of small GTPases such as Rac1 and Cdc42 that lead to enlargement of spines mediated by proteins that in part are found in a complex with the C-terminal domains of Shank3. Alternatively, recruitment of specific Shank3 isoforms with specific domain composition enabling the binding of these factors may occur. In addition, Shank3 recruiting Rich2 to its PDZ domain will facilitate spine enlargement due to an inhibitory effect on the RhoGAP activity of Rich2 (Figure 7).

## 6. Conclusions

The different isoforms of Shank proteins and their interaction partners have a pivotal role in synaptic plasticity via actin remodeling. Because of the different protein-protein interaction domains, Shank can directly and indirectly affect actin dynamics, for example, via small GTPases. This points towards a central physiological role of the synaptic Shank complex in translating synaptic activity in structural and associated changes. Given that actin remodeling is a central motif of Shank signaling, it may not be surprising that the pathomechanisms of ASD associated mutation in Shank proteins also converge on this process. Since the study on small GTPases is much older than the study on Shank, knowledge acquired by small GTPase studies can be at least to some extent useful for the field of Shankopathies.

## Figures and Tables

**Table 1:** Rho GTPase modulators in mental disorders.

Protein	Gene	GTPase signaling	Clinical features	Effect of mutation on spine morphogenesis	Interaction with Shank
Oligophrenin 1	*OPHN1*	GAP for Rac1/Cdc42/RhoA	Intellectual disability, microcephaly, ataxia, hypersensitivity	↓ on spine length and maturation	Indirect via Homer
MEGAP	*MEGAP*	GAP for Rac1/Cdc42	Microcephaly, facial disfigurement	Loss of filopodia and lamellipodia	Not known
RICH2	*ARHGAP44*	GAP for Rac1/Cdc42	No mutation yet identified in human	↑ spine area, multiple spines fused together	Direct
*α*PIX	*ARHGEF6*	GEF for Rac1/Cdc42	Intellectual disability	↓ spine with mushroom morphology	Not known
*β*PIX	*ARHGEF7*	GEF for Rac1/Cdc42	No mutation yet identified in human	↓ synaptic proteins, defective NMJ	Direct
Alsin	*ALS2*	GEF for Rac1, Rab5, Ran	Motor neuron degeneration	↓ axon growth, ↑ cell death	Not known

## Abbreviations

ASD: Autism spectrum disorders
ProSAP: Proline-rich synapse associated protein
PSD: Postsynaptic density
Shank: SH3 domain and ankyrin repeat containing protein
AMPA: *α*-Amino-3-hydroxy-5-methyl-4-isoxazolepropionic acid
NMDA: N-Methyl-D-aspartate
LTP: Long-term potentiation
LTD: Long-term depression
mEPSC: miniature excitatory postsynaptic currents.
